# Getúlio Vargas e o Código de Águas na “reconstrução” do Brasil, 1930-1945: para além da energia e aquém da salubridade

**DOI:** 10.1590/S0104-59702025000100001

**Published:** 2025-02-14

**Authors:** Fábio Alexandre dos Santos

**Affiliations:** i Professor associado, Laboratório de Estudos Interdisciplinares e Análises Sociais/Universidade Federal de São Paulo. Osasco – SP – Brasil. fa.santos@unifesp.br

**Keywords:** Código de Águas (1934), Abastecimento de água, Esgotamento sanitário, Salubridade, Getúlio Dornelles Vargas (1882-1954), Water Code (1934), Water supply, Sanitary sewage, Salubrity, Getúlio Dornelles Vargas (1882-1954)

## Abstract

O objetivo é refletir sobre o Código de Águas de 1934 e os serviços de abastecimento de água e coleta de esgotos no Brasil no primeiro governo de Getúlio Vargas. Tratado pela historiografia majoritariamente como um instrumento para regular o setor de energia hidrelétrica, o código apresenta elementos que o extrapolam: regular o uso da “água” imposto pela conjuntura de acumulação ampliada do capital e sistematizar uma legislação difusa e seus diferentes usos, colocando em evidência questões como salubridade, poluição etc. Tais elementos, por sua vez, correspondem aos planos de Vargas de “reconstruir” a nação. Para tanto, foi realizado um balanço historiográfico sobre os temas e analisada a legislação pertinente.

## Abstract (en)

The objective is to reflect on the Water Code (1934) and the water supply and sewage collection services in Brazil during the first government of Getúlio Vargas. Treated by historiography mostly as an instrument to regulate the hydroelectric energy sector, the code presents elements that go beyond it: regulating the use of “water” imposed by the situation of expanded capital accumulation and systematizing diffuse legislation and its different uses, highlighting issues such as salubrity, pollution etc. Such elements, in turn, correspond to Vargas’ plans to “rebuild” the nation. To this end, a historiographical assessment of the themes was carried out and the relevant legislation was analyzed.

A década de 1930 iniciou-se sob o signo da crise no cenário mundial, marcada essencialmente pelos efeitos da Depressão econômica. Internamente, ao final da década de 1920 ruíram os pilares políticos da Velha República, com Getúlio Vargas assumindo a presidência em 1930; por outro lado, a Depressão afetou a estrutura exportadora brasileira de matérias-primas e alimentos e, consequentemente, a balança de pagamentos. Em meio às instabilidades, Vargas promoveu novos impulsos à industrialização brasileira.^1^

Iniciado em novembro de 1930 como governo provisório, o governo Vargas foi marcado por centralização, regulação e controle – que se intensificaram a partir de 1937 – tanto por meio da elaboração de normativas, criação de novos órgãos e departamentos quanto pela repressão a opositores, como também pela construção da narrativa de salvação da nação. Os polos regionais e oligárquicos de poder foram subordinados ao Estado varguista, que, mesmo assentado sobre o federalismo, se impunha ante os interesses locais por meio de sua estrutura burocrática racional e autoritária (Draibe, 2004, p.54).

Entre suas ações optou-se pelo controle de recursos considerados estratégicos – como água, florestas, mineração, saúde, educação –, os quais foram subordinados à nova fase de acumulação em que se inseria o Brasil. Em síntese, procurou-se instaurar as condições para a reprodução ampliada do capital.

Sob essa conjuntura, o objetivo deste artigo é refletir sobre a relação entre o Código de Águas, de 1934, e os serviços de saneamento (especificamente o abastecimento de água e o esgotamento sanitário), de modo a apreender como as normativas desse código promoveram condições tanto para a institucionalização ulterior de políticas públicas para esses serviços quanto, por extensão, para expressar as intencionalidades de Vargas para a promoção do nacional-desenvolvimentismo, por meio da oferta de serviços públicos, entre eles o saneamento, muitas vezes transpondo seus aspectos físicos (sanitários) e chegando à idealização da “moralidade” útil ao controle do trabalhador. O período de análise se limita ao primeiro governo de Getúlio Vargas, de 1930 a 1945.

Em que pese o debate sobre o grau e a tipologia do nacional-desenvolvimentismo de Vargas, vale ressaltar que o conceito nacional-desenvolvimentista se originou justamente nos anos 1930-1945. Segundo Bielschowsky (2000, p.127-134), suas características essenciais eram a intervenção estatal na economia, por meio de políticas planejadas e investimentos estatais em setores considerados “estratégicos” para a industrialização; a subordinação da política monetária à política de desenvolvimento econômico; e a propensão para políticas de caráter social, ligadas a temas como pobreza, desemprego e atraso cultural. No fundo, acreditava-se na industrialização como forma de superar os atrasos e conquistar a modernidade.

A hipótese aqui delineada é a de que o Código de Águas, ao regulamentar e controlar o uso da água para o setor de geração de energia hidrelétrica, recolocou em perspectiva a água para abastecimento humano, a questão da poluição e da salubridade pública, ampliando as bases para a constituição de políticas de saneamento e para a normatização da gestão dos múltiplos usos das águas, extrapolando o setor energético.

Apreender a problemática em sua totalidade, portanto, implica considerar utilizações e apropriações de recursos como água, solo, florestas, em especial na medida em que tomam a forma de “mercadoria” que, associada a aprimoramentos tecnológicos, científicos e às dinâmicas capitalistas, promovem ressignificações sobre o que se entende por natureza (Worster, 1991, p.201; Martinez, 2006, p.21; Pádua, 2010, p.88). Trata-se essencialmente de considerar a espécie humana como parte constitutiva e indissociável do todo a que se chama de “natureza”, pois, ao mesmo tempo que o indivíduo promove modificações em seu meio, também é por elas afetado. Outrossim, o pilar teórico sobre o qual este artigo se assenta considera que as ações antrópicas para usos e representações dos recursos (aqui chamados de naturais para efeitos analíticos) foram e são alvos de apropriações com fins capitalistas, transformando-os em recursos produtivos, muitas vezes à mercê dos direitos da coletividade.

Os serviços relativos ao que se chama de saneamento básico não fogem a essa premissa. Assim, o setor é tratado nas linhas que seguem, considerando a conjuntura e os elementos constituintes de seu tempo, os quais lhe conferem sentido, ou seja, as interações e intervenções/ações que refletiam as especificidades de sua época e possibilitavam o reconhecimento de sua relevância para a sociedade (Souza et al., 2015, p.14-20; Hespanhol, 2006, p.286).

Reconhecer e implementar políticas públicas para o setor era parte do movimento de assunção, pelo Estado, de sua responsabilidade para com a salubridade, que, se não era um fenômeno novo, era um processo que se assentava sobre novas bases. O Estado, assim, colocava-se em ação, por meio de institucionalizações – administrativas, normativas e operacionais – por meio do que se conceitua como “políticas públicas” (Souza, 2007, p.69).^2^

O Estado, em seus diferentes tempos históricos, não esteve alheio às questões relativas às águas ou à salubridade. Ao contrário, em diferentes momentos o Estado legislou segundo as bases conceituais de que dispunha e, claro, de acordo com os interesses nele acomodados, por vezes, em conflito. No que se refere à oferta dos serviços de abastecimento de água e esgotamento sanitário, especificamente, a partir do século XIX, sua atuação contribuiu para moldar a heterogeneidade que assinala a oferta desses serviços no Brasil, a qual deve ser atrelada às desigualdades historicamente constituídas desde o período colonial, fundado na grande propriedade, no escravismo, na monocultura e nas relações de poder locais (Santos, 2022, 2023), configurando o que Heller (2018, p.134) designa como “padrão de políticas públicas excludentes”.

Captar as dinâmicas da estrutura legal de uma sociedade e os parâmetros por ela determinados é uma das premissas da história ambiental, argumentam Silva (1997, p.212) e Martins (2007, p.37), pois em suas linhas encontram-se as fronteiras normativas para a apropriação e o uso dos recursos naturais. Nessas dinâmicas se manifestam “as práticas econômicas, sancionadas pela lei” (Silva, 1997, p.213), mais precisamente, seria o que Worster (1991) classifica como apreender as dimensões sociais e econômicas que envolvem a milenar inter-relação do humano com os recursos naturais.

O artigo encontra-se estruturado em dois tópicos após esta introdução. No primeiro, apresentam-se alguns elementos do processo de promulgação do código e os interesses envolvidos; no segundo, discutem-se brevemente as formas de se regular a água e o esgotamento sanitário na legislação que antecede os anos 1930 para, então, pensar a relação do código com os serviços de água e esgotos. Por fim, estão as considerações finais. Para tanto, foi realizado um balanço historiográfico sobre os temas que se correlacionam, foi analisada a legislação pertinente, além da consulta a documentos, jornais e periódicos do período.

## O código: interesses conflitantes

Originalmente, o Código de Águas foi proposto em 1907, pelo jurista Alfredo Valladão, a pedido de Miguel Calmon, então ministro da Indústria, Viação e Obras Públicas do governo Afonso Pena, em função de sua publicação, de 1904, intitulada *Dos rios públicos e particulares*, em que debatia a propriedade das águas e a necessidade de discuti-las para além da navegação (Silvestre, 2008, p.9; Saes, 2010, p.231; Pagnoccheschi, 2016, p.179).

A proposta tramitou até ser preterida em 1923 e, a partir de 1924, foi reavivada devido à seca que assolou São Paulo e impactou a geração de energia hidrelétrica (Corrêa, 2005, p.268). Segundo Medeiros (1933, citado em Nunes, 2022, p.241), seria impossível o código ser aprovado na década de 1900, pois se contrapunha à política de descentralização política e às oligarquias regionais, ao passo que o projeto já propunha centralização regulatória no âmbito federal.

Em 1931, sob o governo provisório de Vargas, uma subcomissão legislativa retomou a pauta, estimulada pela nova conjuntura política e econômica.^3^ Para a empreitada foram nomeados o próprio Valladão, que, juntamente com José de Castro Nunes e Inácio Veríssimo de Melo, reescreveu o código (Saes, 2010, p.231; Pagnoccheschi, 2016, p.179; Nunes, 2022, p.239).

Mesmo datado de 10 de julho de 1934, o decreto n.24.643, que estabeleceu o Código de Águas, foi publicado oficialmente – como ato que conclui a sanção de uma lei – em 20 de julho de 1934, portanto, dias após a promulgação da Constituição Federal. Sua publicação se deu envolta em apontamentos políticos e jurídicos que não se dissociavam das críticas ao governo provisório. O jurista José Manuel de Azevedo Marques (1935, p.259)^4^ classificou o Código de Águas como inconstitucional pelo fato de não ter sido apreciado e aprovado pelo Congresso Nacional e não ter sido submetido à análise jurídica, dado que o decreto envolvia direito patrimonial ou individual.^5^ Também criticando o decreto, pronunciou-se o deputado Antônio Augusto de Barros Penteado,^6^ afirmando que, na data de sua promulgação, “sua Mãe, a Ditadura, já não mais exista, tendo cessado de existir desde o dia 17 do mesmo mês, data da publicação da nova Carta Magna; não poderia, pois, dar à luz um filho, uma mãe já morta havia três dias”. Assim, justificava o deputado que encaminhava à Assembleia um projeto de lei para declarar “inexistente” o decreto, inclusive, devido aos erros técnicos do código (Jornal do Commercio, 1934b, p.4).

Até 1938 o Código de Águas ficou suspenso, enquanto perdurou o processo que decidiu pela sua constitucionalidade. Nunes (2022, p.255) aponta a questão demonstrando que a decisão do Supremo Tribunal Federal foi unânime. Entre os argumentos estava a diferenciação entre promulgação, para validar a norma, e a publicação, visando dar publicidade à decisão. O embasamento foi que a forma normativa “era de decreto, [assim] estes seriam de aplicação imediata, pois ato fruto de um único poder (Executivo), diferente das leis que são um ato coordenado entre o Legislativo (que decreta) e o Executivo (que promulga)”.

Em outra frente, as empresas estrangeiras que atuavam no setor elétrico ampliaram o coro contra o código. Somavam-se a ele insatisfações decorrentes de dois decretos de 1931: um que proibia transações ou transferências de cursos e quedas d’água, pois entrava em discussão o Código de Águas,^7^ e o segundo extinguiu a cláusula ouro, que permitia às empresas atrelarem seus contratos ao ouro. Em essência, as empresas perderam o colchão que as protegia das variações cambiais a que estavam sujeitas com as importações de máquinas e equipamentos. Caso exemplar foi o da Light and Power, que gerava e distribuía energia nas cidades de São Paulo e Rio de Janeiro (Corrêa, 2005).

Entretanto, havia mais uma questão que reforçava a oposição da Light ao código, relacionada aos capítulos IV e V, que estabeleciam a linha média das inundações para efeitos de desapropriação de terras. Na ocasião, a Light encontrava-se em batalha jurídica com proprietários de terras pelo direito de desapropriação das áreas atingidas pela enchente de 1929, em São Paulo. Segundo a empresa, deveriam ser obedecidas as prerrogativas da Lei de Concessão de 1927, que a autorizou retificar e inverter o curso do rio Pinheiros para geração de energia hidrelétrica na usina de Cubatão.^8^ Nela constava o direito de desapropriação de toda a área inundada. Nesse caso prevaleceu a lei de 1927.

Segundo McDowall (2008), que historiciza a atuação da empresa pelo olhar e interesse da multinacional, no enfrentamento ao Código de Águas a Light procurou ganhar tempo por meio do que classificou como “política do silêncio”, associando-se a opositores na batalha que se seguiu. Contudo, para a Light, conclui McDowall, “infelizmente ... o Código de Águas nunca diminuiu a ponto de desaparecer” (p.414). Outras duas associações que se juntaram contra o código foram a Sociedade Rural do Triângulo Mineiro, de Uberaba (MG), por intermédio de seu presidente, o ex-deputado Fidelis Reis, e a Associação Comercial de São Paulo, na pessoa de Alcântara Machado. O primeiro declarava que dificilmente o código permitiria investimentos “onde a escassez de capitais tem exigências mais liberais” (Jornal do Commercio, 1934a, p.7). O segundo, que a regulamentação impunha dificuldades para as empresas de energia devido à impossibilidade de se contratar financiamentos e, portanto, impactando a geração e a oferta de energia, ao passo que aumentava sua demanda. Solicitava, assim, que o projeto de lei apresentado por Barros Penteado recebesse a atenção que acreditava merecer (Jornal do Commercio, 1934c, p.4).

Até então, estados e municípios detinham o poder de conceder a exploração de recursos naturais (entre eles, as águas, as quedas e os cursos) e negociar contratos sem interferência da União. Na prática, portanto, o Código de Águas subtraiu o poder de ação de estados e municípios, regulando os serviços e as tarifas, mas, por extensão também regulando o uso e a exploração de nascentes, quedas e cursos d’água etc.

A despeito das atenuações no código que se seguiram e da criação, em 1939, do Conselho Nacional de Águas e Energia Elétrica (CNAEE), que passou a gerir as águas para geração de energia, ele encontra-se em vigor na atualidade e tem sido objeto de inúmeras pesquisas. Entre as principais estão as que focam nas águas pelo setor de energia, tanto do ponto de vista econômico (Bastos, 2012, p.276-277; Espósito Neto, 2015, p.7) quanto jurídico (Carvalho et al., 2008, p.113) e, em alguns casos, estendendo-as para o legado que deixaram para a ulterior legislação sobre os seus múltiplos usos e a concentração da gestão nas mãos da União (Pagnoccheschi, 2016; Mourad, Rodrigues, 2019; Daronco, 2013; Dias, Nunes, 2020; Santos et al., 2021). No sentido dessa reflexão, Silvestre (2008) aponta que o código atendeu à acumulação capitalista e que, a despeito das prerrogativas de a água ser tratada pela sua essencialidade à vida, prevaleceram os interesses das companhias hidrelétricas.

Isto posto, parece claro que, no centro do debate, estavam interesses e impactos sobre o setor de energia, além de questões econômicas que pairavam sobre o controle dos recursos naturais necessários à reprodução ampliada do capital. Aí encontra-se a vinculação com a salubridade, com a saúde, com as águas para abastecimento e esgotamento, pois, como pronunciou Vargas (1941, p.244), “sanear, educar e povoar não podem ser resultado espontâneo de atividades dispersas e quase sempre dispersivas. Torna-se necessário dar-lhes unidade de ação para alcançar os objetivos visados”. Unidade em que convergiam questões políticas, econômicas e de infraestrutura da salubridade para a “reconstrução da nação”.

Nesse sentido os conservacionistas,^9^ que há décadas vinham refletindo sobre proteção dos recursos naturais, mobilizaram-se em favor dos códigos. Historicamente suas ideias foram discutidas sob diferentes concepções, alinhados aos axiomas de cada “tempo” – entre eles cronistas, viajantes, intelectuais, cientistas, médicos, engenheiros e sanitaristas, dando concretude à temática no início do século XX. Segundo Dean (1996), institutos, sociedades, clubes etc. foram criados desde o início do século XIX, e, em meados daquele século, algumas vozes já associavam o desmatamento às recorrentes secas ou rios que, antes caudalosos e estáveis, cada vez mais carregavam lama e abrigavam bancos de areia. Contudo, antes de 1930, essas congregações desempenharam tímido protagonismo junto ao Estado, mesmo algumas tendo origem ou recebido apoio estatal.

Entre aqueles grupos estava a Sociedade dos Amigos das Árvores, que convocou a primeira Conferência Brasileira de Proteção à Natureza, em 1934 (Sampaio, 1935a, 1935b; Dean, 1996; Franco, Drummond, 2009). Seus idealizadores foram capazes, a sua maneira e dentro dos seus limites, de apoiar e influenciar o governo Vargas na formulação de políticas conservacionistas ao associarem proteção à natureza com a construção da identidade nacional. Pouco antes da conferência, Alberto José Sampaio^10^ declarou que as normatizações do governo provisório, de missões artísticas e científicas, de caça e pesca e até o Código Florestal, eram evidências da “atenção dos poderes públicos” sobre a necessidade de proteção da natureza (Sampaio, 1934, p.33).

Realizada entre 8 e 15 de abril de 1934, com patrocínio do governo Vargas, e relatada por Sampaio, os conferencistas^11^ intencionavam “pressionar” o governo para a instauração de políticas que resguardassem a natureza (Franco, Drummond, 2009, p.43), em três pontos essenciais: a defesa dos recursos naturais e da paisagem; a necessidade de o Brasil acompanhar os debates internacionais sobre a defesa da natureza e contra o desmatamento; e as suas implicações para a fauna e flora, o que incluía a defesa dos mananciais visando ao abastecimento humano e à regulação do clima.^12^

Leôncio Corrêa, presidente da Sociedade dos Amigos das Árvores, assim declarou na abertura da conferência: “O problema florestal é, ao mesmo tempo, um problema econômico, um problema social, de higiene, de riqueza, de importância capital e de relevante transcendência”. Dessa forma, explicitou seu apoio ao Código Florestal, publicado meses antes (citado em Sampaio, 1935a, p.15-19).

Quanto ao Código de Águas, o professor Saladino Gusmão apontou a necessidade de aprová-lo, junto com a imperiosa proteção das cabeceiras dos rios e mananciais (citado em Sampaio, 1935a, p.37). Assim como os demais conferencistas, o professor Durval Ribeiro do Pinho indicava as árvores como responsáveis por processos fundamentais para a sociedade: “As árvores são também indispensáveis à saúde: melhoram o clima e o tornam salubre. ... Não é simples questão de embelezamento mas uma medida indispensável à higiene e salubridade pública” (citado em Sampaio, 1935a, p.41).

Do ponto de vista mais amplo dos códigos, Franco (2002, p.94) afirma que os conservacionistas trataram de endossar as normativas de 1934 relativas ao controle dos recursos naturais, na medida em que a legislação contemplava o reconhecimento e a proteção dos recursos e das belezas naturais. Entretanto, ressalvavam que tais políticas deveriam ir além da normatização, abarcando a educação, tanto econômica quanto estética e científica, o uso eficiente dos recursos e a instauração de áreas de proteção, como parques e reservas florestais.^13^

Em síntese, diante de interesses dissonantes, as normatizações visavam controlar a apropriação e a exploração dos recursos naturais, reforçando o poder da União ante os intentos locais e, por vezes, do capital. No caso do Código de Águas, mesmo direcionado ao setor de energia, suas prerrogativas colocaram em perspectiva outros usos da água, expandindo sua aplicabilidade, os debates e os conflitos.

## Água e esgoto: antes e depois do Código de Águas

Até os anos 1930, a legislação que tratava das águas era esparsa e difusa. Historicamente, estava embasada no Direito Romano, nas Ordenações do Reino e no Alvará de 1804, os quais pontuavam as diretrizes sobre o regime dos rios e o uso das águas (Valladão, 1904, p.13).

Em resumo, as prerrogativas legais como colônia de Portugal centravam-se essencialmente na navegação e na pesca, isto é, em aspectos econômicos e militares (defesa), porque confluíam estrategicamente para a manutenção do poder territorial, político e econômico; ao passo que as soluções para o consumo humano da água e o descarte de resíduos eram individuais, este, na maioria das vezes, executado por escravos. Algumas localidades, ao longo do século XIX, ampliaram a oferta de água instalando chafarizes, outras concederam os serviços a empresas privadas, na maioria dos casos, de capital estrangeiro.

Na primeira Constituição sob a República, de 1891, a posse das águas se manteve atrelada à propriedade do solo, assim como previa a Constituição de 1824 (Brasil, 1824), prevendo a indenização ao proprietário no caso de necessidade e utilidade pública, segundo o art. 72 em seu parágrafo 17 (Brasil, 1891). Ao mesmo tempo, contudo, ampliou-se a descentralização dos serviços públicos num momento importante para os estados mais bem posicionados em termos de participação na balança comercial, pois, ao arrecadarem recursos, viabilizavam investimentos em serviços públicos ou, ainda, encampações de empresas de saneamento existentes, como em São Paulo, Porto Alegre, Pelotas, Rio Grande e Manaus, amparadas pelas reclamações ante a carência de atendimento e/ou a má qualidade dos serviços. Também se promulgou a descentralização das organizações sanitárias, conforme decreto federal de 30 de dezembro de 1891, autorizando os estados a organizarem seus respectivos serviços desde que se restringissem às áreas terrestres, pois ao governo federal competia a responsabilidade sanitária dos portos e das embarcações marítimas.^14^

As águas potáveis, especificamente, receberam atenção no Código Penal de 1890, considerando sua degradação, impetrando a possibilidade de pena de prisão ao poluidor no caso de torná-la nociva à saúde humana. Já o Código Penal de 1916 tratou-as do ponto de vista do direito de uso, do direito de vizinhança e de sua utilização pela premissa da propriedade, reafirmando o valor econômico das águas (Pagnoccheschi, 2016; Santos et al., 2021; Souza, Tavares, 2021).

Quanto aos serviços de abastecimento de água e esgotamento sanitário, na Constituição de 1934, ambos estavam vinculados à saúde. Não que devessem necessariamente estar desvencilhados quanto às suas finalidades, claro, porém, inexistiam especificidades que poderiam denotar os escopos de cada um dos serviços, isto é, os meios (políticas) para a promoção de seus objetivos comuns.

Competia à União e aos estados, por exemplo, cuidar da saúde e da assistência públicas, mas sem defini-las, conforme o art. 10 apregoava; mais adiante, o art. 121 assinalava que à legislação cabia o “amparo” da produção e das condições de trabalho, “na cidade e no campo”, visando à “proteção social do trabalhador e [a]os interesses econômicos do país” e, ainda, à “assistência médica e sanitária do trabalhador” (Brasil, 1934d). Ao reforçar as determinações relativas à assistência e à saúde, o artigo ia além ao incorporar as condições físicas dos trabalhadores, atrelando-as à necessidade de um trabalhador “sadio” e “capacitado” para atender às condicionalidades econômicas do país.

E prossegue a Carta Magna, em seu art. 138, definindo as competências da União, dos estados e dos municípios, amparando-se, inclusive, em noções eugênicas, classificadas como forma de se opor as ameaças à moralidade social:

b) estimular a educação eugênica; ... f) adotar medidas legislativas e administrativas tendentes a restringir a moralidade e a morbidade infantis; e de higiene social, que impeçam a propagação das doenças transmissíveis; g) cuidar da higiene mental e incentivar a luta contra os venenos sociais (Brasil, 1934d).^15^

O que se nota, assim, é uma profusão de peças legislativas – características de cada tempo –, mas ainda insuficientes para amparar, regular e direcionar a gestão das águas e as suas diferentes utilizações para aqueles anos, enquanto inexistiam políticas nacionais para o saneamento.

Com o Código de Águas de 1934, o caráter econômico conferido às águas foi ressignificado. A partir de então, a regulamentação pretendida considerava a água como insumo fundamental para a geração de energia (hidrelétrica) sob a conjuntura da convergência do deslocamento do centro dinâmico da acumulação (Furtado, 1963). O próprio preâmbulo do código é explícito em suas intencionalidades: o controle e o incentivo ao “aproveitamento industrial das águas ... que facilitem e garantam seu aproveitamento racional” (Brasil, 1934a).

Entre as normativas que remetem especificamente ao abastecimento de água e/ou ao esgotamento sanitário, o art. 34 assegura o acesso gratuito à água para consumo humano em “qualquer corrente ou nascente de água, para as primeiras necessidades da vida”, incluindo as áreas territoriais com fontes e os seus acessos particulares desde que respeitados o direito de propriedade do espaço. Reafirmando a disposição, estabeleceu que, no uso das águas públicas,^16^ caberia a “preferência” para o abastecimento das populações, estando estas sujeitas à regulamentação administrativa da localidade em que se situavam (art. 36), reiterado pelos artigos 71 e 72, ao destacar a proibição de desvios, quando um curso d’água atender ao abastecimento de populações; e, ainda, reforçado pelo art. 94, que, mesmo classificadas como particulares, não poderiam ser desviadas se abastecessem a população.

Outro aspecto é a preocupação com a “poluição” e a “contaminação” das águas. Mesmo não sendo novidade na legislação, o código parece ampliar as especificações sobre o tema. O art. 98 indica proibições de construções capazes de “poluir ou inutilizar” águas de poços ou nascentes. O capítulo VI, intitulado “Águas nocivas”, em seu art. 109, denota o aspecto criminal de possíveis contaminações, assim como no Código Penal de 1916:

A ninguém é lícito conspurcar ou contaminar as águas que não consome, com prejuízo de terceiros.

Art. 110. Os trabalhos para a salubridade das águas serão executados à custa dos infratores, que, além da responsabilidade criminal, se houver, responderão pelas perdas e danos que causarem e pelas multas que lhes forem impostas nos regulamentos administrativos (Brasil, 1934a).

Entrementes, no artigo seguinte, o código abre uma brecha para “inquinar” as águas, desde que autorizadas, para atender aos “interesses relevantes” da agricultura ou da indústria. Nesse caso, seus poluidores deveriam cuidar da sua purificação “por qualquer processo, ou sigam o seu esgoto natural”, sem especificar do que se trata esse “sigam o seu esgoto natural”. As penalidades para o descumprimento incluem indenizações e multas.

Paralelamente, o código destaca que os terrenos “pantanosos, quando, declarada sua insalubridade”, deveriam ser “dessecados” pelos proprietários, e, se assim não o fossem, seriam executados pela administração pública, a qual teria o direito de receber uma “taxa de melhoria sobre o acréscimo do valor dos terrenos saneados”; em caso de descumprimento, ocorreria a “desapropriação, indenizado o mesmo na correspondência do valor atual do terreno, e não do que este venha a adquirir por efeito de tais trabalhos”, ou seja, da valorização correspondente aos serviços (artigos 112 a 116).

Contudo, sob o ponto de vista do abastecimento de água e do esgotamento sanitário, a normativa alcançou um patamar mais amplo, na medida em que incorporou os múltiplos usos da água, as suas possibilidades e os seus limites, e introduziu de forma articulada a esses usos a problemática da contaminação e as consequências para a coletividade. O art. 143 é emblemático nesse sentido:

Em todos os aproveitamentos de energia hidráulica serão satisfeitas exigências acauteladoras dos interesses gerais:

- da alimentação e das necessidades das populações ribeirinhas;
- da salubridade pública;
- da navegação;
- da irrigação;
- da proteção contra as inundações;
- da conservação e livre circulação do peixe;
- do escoamento e rejeição das águas (Brasil, 1934a).

Assim, em termos legais, parece evidente a pretensão de submeter a utilização das águas a fatores relacionados à salubridade pública, o que deixa entrever os serviços de abastecimento de água e esgotamento sanitário.

Utilizando as terminologias do próprio código, em especial para dimensionar suas premissas conceituais, cabe reter a significância do verbo acautelar, remetendo para setores como o de saneamento, ou seja, para proteger, acautelar outros usos e populações nos seus diversos interesses. Assim, a impressão é a de que o uso coletivo e múltiplo da água emerge como um elemento fundamental do código.

Tais disposições acauteladoras encontraram ressonância nas críticas do deputado Barros Penteado, para quem “não deveriam figurar” no decreto, pois, segundo ele, “o concessionário [de energia] não poderá iniciar a construção das obras sem a aprovação prévia do plano geral, no qual deverão estar previstas as hipóteses figuradas neste item” (Jornal do Commercio, 1934b, p.4), estando sujeito à caducidade do empreendimento, conforme o art. 168, remetendo à possiblidade de infração segundo os artigos 143 e 153, que prezam pelas águas necessárias ao funcionamento dos serviços públicos.

... o poder concedente não aprovará o plano das obras se dele se verificar que a derivação pode transportar maior volume de água do que a concedida, se essas obras projetadas prejudicam a salubridade pública, concorrem para inundações, dificultam a navegação etc. (Jornal do Commercio, 1934b, p.4).

Em essência, estava a disputa pelo uso das águas, entre o setor de energia e o interesse público. Para Silvestre (2008), o código priorizou a regulamentação das ações que pudessem obstruir o livre curso d’água para geração de energia, já que a geração hidrelétrica depende da regularidade no seu fluxo. Para a autora, portanto, o abastecimento de água não foi priorizado. Talvez pela primeira vez uma única peça regulatória tenha colocado em perspectiva diferentes usos e finalidades para a água, o que não deve ser negligenciado. Ademais, o Código de Águas não pode ser tomado isoladamente, ao contrário, deve ser compreendido em conjunto com as normativas publicadas naquele ano, com as institucionalizações e os acordos de cooperação promovidos antes e depois de 1934 à luz dos debates sobre saneamento existentes desde a década de 1910 e das discussões sobre proteção dos recursos naturais.

Com relação a outros códigos, o Código Florestal também intentou impor um caráter racional para o uso e a gestão dos recursos florestais, destacando os fins econômicos das florestas sob a perspectiva da ampliação da demanda por energia (lenha) e por matérias-primas para a indústria, além de dissociar, assim como nos Códigos de Águas e de Minas, a propriedade do solo dos recursos naturais. Na relação com as águas, seu art. 4^o^ especifica a função das florestas classificadas como protetoras, as quais teriam a função de preservar o regime das águas, os mananciais e os cursos d’água, por meio do trato das matas ciliares e da vegetação “protetora”, assim como assegurar as condições de salubridade pública (Brasil, 1934b). Ao passo que era proibido aos proprietários do solo, das florestas, o uso de fogo em campos ou áreas de preparação para lavoura ou em locais de vegetação escassa, em especial às margens de cursos d’água (art. 22).^17^

Já o Código de Minas, ao tratar da água, considerou em seu art. 67 a possiblidade de contaminação de cursos d’água em função dos trabalhos da lavra, preconizando que, se as “águas dos mananciais, dos córregos ou dos rios forem poluídas”, caberia ao Estado averiguar as ocorrências e tomar as medidas cabíveis “para evitar os males públicos, em vista, quanto possível, as condições econômicas da lavra da mina” (Brasil, 1934c). Ambos os códigos também foram considerados ganhos à sociedade pelos conservacionistas, como apontado.

As parcerias, os convênios e as associações contemplam parte da totalidade em análise. Na década de 1910, importantes convênios com entidades ou organizações voltadas ao combate a endemias já haviam tomado forma no Brasil, como o acordo de cooperação com o International Health Board, da Fundação Rockefeller, em 1917, que aplicou no país sua experiência de combate à ancilostomíase, no Sul dos Estados Unidos, sugerindo, entre outras ações, a criação de postos sanitários que promovessem educação sanitária e saneamento, com instalação, uso e conservação das fossas e latrinas (Costa, 1986, p.114; Palmer, 2015).

Nos anos seguintes, a fundação firmou convênios com estados e ampliou sua parceria com o governo federal, atuando no ensino, na formação de profissionais de saúde, na realização de pesquisas e publicações e, ainda, intensificou sua atuação no combate à febre amarela. Segundo Hochman (1998, p.104), a fundação legitimou o movimento para o saneamento rural que se seguiu, com a Liga Pró-Saneamento do Brasil, de 1918, que visava transformar o trabalhador doente e cansado em elemento produtivo e sadio, apto ao trabalho. Ambos legaram ações, reflexões e experiências para ulteriores políticas para os setores de saúde e saneamento (Costa, 1986; Hochman, 1998; Löwy, 1999; Rezende, Heller, 2002; Cueto, 2015).

O que se evidencia, portanto, é uma convergência de iniciativas pretéritas com matizes políticas, econômicas e científicas em desenvolvimento desde o início do século XX, as quais desencadearam discussões e políticas para o aproveitamento dos recursos naturais por parte do governo Vargas, especialmente porque dialogavam de alguma forma com a almejada reconstrução da nação. Entretanto, a noção de proteção à natureza que permeia as linhas das institucionalizações de Vargas subordinava os recursos naturais a aspectos racionais e utilitaristas, cujo determinante não era proteger a natureza *per se*, mas o desenvolvimentismo.

A coalizão de poder formada em 1930 se revelou solidamente desenvolvimentista, no sentido menos ‘ambiental’ que o termo pode assumir. Mesmo sofrendo diversas modificações em 1937, 1945 e 1964, o desenvolvimentismo oficial brasileiro expressou uma visão largamente disseminada na sociedade de que o crescimento econômico deveria ser perseguido a todo custo, inclusive, mas não exclusivamente, os custos ambientais (Franco, Drummond, 2005, p.159).

Do ponto de vista administrativo, o governo Vargas institucionalizou órgãos, departamentos e diretorias com o objetivo de estruturar os serviços de saúde e saneamento, entre eles o Ministério da Educação e Saúde Pública (Mesp), em 1930 (regulamentado em 1934) e, em 1935, renomeado como Ministério da Educação e Saúde (MES). Mas foi a partir de 1937, sob o Estado Novo, que reestruturações significativas tomaram forma no âmbito do MES e dos serviços sob sua responsabilidade. Segundo Corrêa (2004, p.232), suas ações, assim como de outros órgãos do governo, espraiavam as premissas do “amor à Pátria, o otimismo quanto ao poder e o destino de nossa raça”.

Sob a direção de Gustavo Capanema (1934 a 1945), o Departamento Nacional de Saúde Pública (DNSP), de 1920, foi substituído pelo Departamento Nacional de Saúde (DNS), e, em 1941, a Diretoria Nacional de Saúde e Assistência Médico-Social (DNSAMS) passou a controlar os departamentos estaduais de saúde, numa ação tipicamente centralizadora. Em 1941, ainda, ocorreu a primeira Conferência Nacional de Saúde, quando o Estado ouviu delegados de todo o país para as áreas de educação sanitária, fiscalização da medicina e saúde dos portos, serviços de bioestatística e o serviço federal de águas e esgotos. Estes últimos serviços ficaram nas mãos dos interventores nos estados, apresentando resultados pífios (Rezende, Heller, 2002, p.176).

Outra instituição emblemática foi Serviço Federal de Águas e Esgotos (SFAE), que, embora tivesse atribuições para todo o Brasil, se restringiu ao Distrito Federal, e anos depois foi transformado em Departamento de Água e Esgotos do Distrito Federal. Rezende e Heller (2002, p.176) sugerem que o SFAE teve sua origem como suporte ao programa de saneamento do Amazonas, chegando a elaborar projetos de abastecimento e esgotamento sanitário para a região Norte, mas as ações previstas ficaram no papel. Em seguida, o Serviço Especial de Saúde Pública (Sesp) assumiu o programa de saneamento da Amazônia.

A atuação do Sesp no conjunto da implementação das políticas de saneamento foi emblemática. Criado em 1942, sua gênese remete à conjuntura da Segunda Guerra Mundial e aos interesses dos norte-americanos por recursos minerais e vegetais, como borracha, estanho, quinino e fibras, os quais eram encontrados em regiões do vale do Amazonas, no estado de Goiás e no vale do rio Doce. Denominado de “contrato básico” entre Brasil e EUA, o Sesp estava subordinado ao Instituto de Assuntos Interamericanos (Iaia), sob a responsabilidade do MES, mas com autonomia jurídica e desvinculado das prerrogativas do DNS (Rezende, Heller, 2002, p.180; Lima, 2002, p.46; Campos, 2006, p.17; Menicucci, D’Albuquerque, 2018, p.12; Dias, Nunes, 2020, p.11).

Na origem do acordo foram contemplados interesses múltiplos de ambos os países, desde militares, com a cessão de base militar aos norte-americanos no Nordeste com a contrapartida de o Brasil receber armamentos americanos, passando por acordos econômicos, até o fornecimento de técnicas e conhecimentos de saúde pública aos brasileiros, em discussão internacionalmente.

Para o setor de abastecimento de água e esgotamento sanitário, especificamente, argumenta Campos (2006, p.26), as ações do Sesp estavam alinhadas às políticas de interiorização e expansão dos serviços do Estado varguista, com a educação sanitária, a construção e administração de sistemas de água e esgotos; além, é claro, do combate a endemias, da construção e gestão de equipamentos sanitários, escolas de enfermagem, hospitais e centros de saúde, normatizações, convênios com estados e municípios; formação de mão de obra. Até 1949, o Sesp executou obras sanitárias em várias cidades, como Itacoatiara, Parintins (AM); Abaetetuba, Macapá, Santarém, Cametá e Marituba (PA), na Amazônia; Aimorés e Governador Valadares (MG); e Colatina (ES), no vale do rio Doce, as três últimas com água e esgoto (Ministério da Saúde, 1972, p.34).

Menicucci e D’Albuquerque (2018, p.12) apontam que, por meio das ações do Sesp, houve “avanço do saneamento com a construção e financiamento de sistemas sanitários”. Lima (2002, p.47), por sua vez, indica que a atuação dos norte-americanos por meio de agências e parcerias com os brasileiros gerou um impacto para o sanitarismo brasileiro, ao mesmo tempo que fez do Brasil um grande laboratório de aprendizagem sobre saúde pública para os norte-americanos aplicarem os conhecimentos aqui adquiridos em outras regiões do planeta. Já Costa (1994 citado em Rezende, Heller, 2002, p.181) aponta que a primeira fase de atuação do Sesp foi marcada pelo detrimento das ações de saneamento e educação sanitária em nome de práticas curativas, com uso de quimioterápicos e biocidas, só se reconfigurando após 1949. Essa nova fase foi marcada pela renovação do convênio e por ações de caráter “nacionalista” (Rezende, Heller, 2002, p.195).

Cabe sublinhar que, ao se prorrogar o convênio e o financiamento norte-americanos, foi possível a ampliação de convênios com estados e municípios, com destaque para a criação da Comissão de Desenvolvimento do Vale do São Francisco, de programas de saneamento e a proposição de um modelo de gestão para os serviços de abastecimento de água e esgotamento sanitário por meio de autarquias (Rezende, Heller, 2002, p.195).

A partir de críticas à burocracia, à dependência de recursos orçamentários e ao caixa único, característico da administração direta, foram constituídas as primeiras autarquias de saneamento, objetivando maior autonomia desses serviços. Assim, surge uma diretriz para o setor saneamento, que é a autossustentação tarifária e o financiamento com recursos onerosos para a implantação de sistemas de água no Brasil. Em 1952, foram instituídos os serviços autônomos de água e esgotos, os SAAEs, originalmente proposto pelo Serviço Especial de Saúde Pública ... Algumas repartições ou inspetorias passaram a ser chamadas de departamento, na forma de autarquias. Em 1953, foi criado o primeiro plano de financiamento federal para abastecimento de água, que teve no Sesp órgão técnico assessor (Risi Junior, Nogueira, 2002, p.140).

Ao discursar sobre os dez anos de governo durante um banquete, em 11 de novembro de 1940, oferecido pelas “classes conservadoras e trabalhistas”, Vargas enfatizou essas relações exaltando diversos temas caros ao seu governo e, entre eles, estavam os que aqui estão em análise:

Existiam em 1930, 90 açudes, com capacidade para 120 milhões de metros cúbicos dágua. A partir de 1931, construíram-se 29 reservatórios, com um total de acumulação de l bilhão e 250 milhões de metros cúbicos, ou sejam 68% do total ora existente. As áreas irrigadas atualmente atingem 5.000 hectares, em 6 redes de canais, isto é, o quíntuplo do que havia naquele ano. ...

Possui igual alcance econômico o saneamento das terras baixas do Estado do Rio. Essa vasta região, antes abandonada e inabitável, está sendo transformada no celeiro natural da metrópole brasileira. Foram saneados 3.000 quilômetros quadrados de terras, em grande parte, já ocupados por culturas produtivas, e [em] breve ficarão prontos mais 1.700, além de 4.000 quilômetros de rios desobstruídos.

A inversão de dinheiros públicos nessas obras soma algumas centenas de milhares de contos e virá beneficiar a economia geral e a saúde de mais de 2 milhões de brasileiros.

...

Temos procurado combater, sem medir sacrifícios, todas as causas de depauperamento do homem e das suas resistências físicas. ...

As obras de saneamento e abastecimento dágua têm sido ampliadas em todos os centros urbanos do país, destinando-se-lhes recursos especiais, o que muito contribui para a melhoria das condições higiênicas gerais. Também, nos últimos anos, além dos serviços novos de educação e higiene, promoveu-se incansável campanha para a boa alimentação popular, e, no setor das indústrias, instalam-se refeitórios capazes de alimentar os trabalhadores, sadiamente e por preços módicos. Generalizam-se, igualmente, os cuidados pela saúde infantil. Fundaram-se centros de puericultura, e instituiu-se o amparo legal à família, visando estimular as proles numerosas, em benefício da higidez da raça e da extensão do povoamento nacional (Vargas, 1941, p.171-174).

Ao final do primeiro governo Vargas, a ambição de instituir nacionalmente serviços para a “reconstrução da nação”, como o abastecimento de água e o esgotamento sanitário, não atingiram o objetivo almejado. Segundo Souza et al. (2015, p.42), ao longo da década de 1940, nas cidades médias e grandes, a oferta domiciliar da água chegou a 40% da população; contudo, tal serviço ainda era inexistente na maioria das localidades. Já Pagnoccheschi (2016, p.180) estima que somente 30% da população era atendida com serviços de abastecimento no Brasil nos anos 1940.

Por fim, mesmo diante de institucionalizações, convênios de cooperação, influências conservacionistas etc., os recursos naturais continuaram sendo largamente apropriados e explorados; da mesma forma que arrefeceu o reduzido protagonismo conservacionista sobre o governo. Segundo Franco e Drummond (2009, p.218), prevaleceu o desenvolvimentismo. Da mesma forma, preocupações com a salubridade (incluindo abastecimento de água e esgotamento), que foram além com o Código de Águas, na prática, não foram suficientes para atender à demanda crescente.

## Considerações finais

Como se procurou demonstrar, o primeiro período de Vargas na presidência foi marcado pela presunção de se “reconstruir a nação”. Para tanto, instituiu, legal e administrativamente, as bases de sustentação para galgar tal objetivo em prol do nacional-desenvolvimentismo, amparado em ações que se caracterizaram por centralização, regulação e controle, inclusive ditatorial.

Dois aspectos são destaques na análise. O primeiro diz respeito a um espectro mais amplo referente ao que se categorizou, aqui, como recursos naturais: florestas, minas, pesca e, especialmente, as águas. Tanto as duas Cartas Magnas dos anos 1930 quanto as diferentes normativas publicadas no período trataram-nos como recursos estratégicos para a economia, cabendo a responsabilidade e o controle ao Estado. Sob essa premissa, a ênfase recaía em suas condicionalidades jurídicas para apropriação e utilização e, com menor ênfase, nos aspectos relativos à proteção dos recursos naturais, como demandavam os conservacionistas, mesmo convergindo com os ideais de Vargas. Em essência, as ações de Vargas se opunham ao liberalismo por meio do controle que, por sua vez, confrontava interesses locais.

O segundo aspecto se refere especificamente ao Código de Águas e à sua relação com os serviços de abastecimento de água e esgotamento sanitário. Por mais que se reconheça que sua ênfase tenha sido no setor de geração de energia hidrelétrica, suas prerrogativas o ultrapassaram. Em primeiro lugar, reafirmou a noção do acesso à água como direito, sem desconsiderar o direito de propriedade das áreas com fontes, mananciais ou cursos d’água, e, assim como a Constituição de 1934 e a de 1937 (Brasil, 1934d, 1937), desvinculou o recurso “água” da propriedade do solo, com “preferência” para o abastecimento das populações, mesmo que sujeitando as fontes à regulamentação local.

A problemática da poluição ou “contaminação” das águas foi outro item importante, ao recolocá-la em correlação com outros usos, reforçando seu caráter criminal, ao passo que, contraditoriamente, abria brechas para “inquinações” autorizadas administrativamente para atender a interesses econômicos, isto é, desenvolvimentistas. Talvez seja o art. 143 o mais paradigmático, na medida em que submeteu o aproveitamento hídrico para geração de energia ao resguardo de outros usos e implicações: para o consumo humano, alimentação, salubridade, irrigação, contra inundações, despejo de resíduos, navegação. Apesar do avanço, os recursos naturais continuaram subordinados aos interesses do capital.

É inegável que o Código de Águas consolidou questões jurídicas para o setor de energia, regulamentadas ao longo dos anos, mas também assentou bases importantes para discussões sobre salubridade e saneamento. Suas diretrizes, além de incorporar e expressar discussões iniciadas há décadas, também devem ser pensadas em conjunto com as ações executadas por instituições do Estado e entidades conveniadas, por mais que a carência dos serviços tenha se mantido. Isto posto, mesmo com sua publicação sob o varguismo e a conjuntura do deslocamento do centro dinâmico da acumulação, deve, então, ser compreendido a partir de uma multiplicidade de vozes e das necessidades que historicamente estiveram envolvidas com a saúde e o saneamento. Portanto, o Código de Águas foi além do setor de energia, mas aquém de sua demanda na área de saneamento.

## Data Availability

Não estão em repositório.

## NOTAS

1 Entre as principais referências historiográficas sobre a Era Vargas estão Carone (1976); Fonseca (2003); Bielschowsky (2000); Corsi (2002); Draibe (2004); Bastos (2012).

2 Estado este destituído de neutralidade (Bresser-Pereira, 2016, p.6). Ele é sistematizador do interesse político da “sociedade civil”, cujos agentes podem ou não estar nele alocados, mas com certeza terá nele direcionamentos oriundos dos interesses dominantes da sociedade de classes, com poder sobre o território e outros importantes níveis da realidade (Gramsci, 2017; Mendonça, 2014; Freitas, 2015).

3 Sobre este processo ver, em especial, Nunes (2022).

4 José Manuel de Azevedo Marques (1865-1943) bacharelou-se em direito, foi deputado estadual e federal pelo Partido Republicano Paulista (PRP), docente na Faculdade de Direito de São Paulo e, ainda, ministro das Relações Exteriores nomeado pelo presidente Epitácio Pessoa.

5 O decreto n.24.642 que estabeleceu o Código de Minas também data de 10 de julho de 1934, com publicação no *Diário Oficial da União* (DOU) em 20 de julho de 1934 (Brasil, 1934c). Já o decreto n.23.793, que estabeleceu o Código Florestal, foi publicado no DOU em 9 de fevereiro de 1934 (Brasil, 1934b).

6 Antônio Augusto de Barros Penteado (1876-1958) era de tradicional família cafeicultora paulista. Participou com a família da Revolução Constitucionalista de 1932, foi deputado constituinte em 1933-1934 por São Paulo e um dos fundadores do Partido Constitucionalista. Também atuou em várias empresas de energia.

7 Similarmente, a suspensão também ocorreu para exploração das jazidas minerais.

8 Sobre a Light (empresa de geração e distribuição de energia nas cidades de São Paulo e Rio de Janeiro), sua atuação e suas intervenções nos cursos d’água, em especial no rio Pinheiros, ver Seabra (1987) e Santos (2011).

9 Na atualidade categoriza-se como “ambientalistas”.

10 Alberto José Sampaio (1881-1946) era botânico e médico, com interlocução científica no Brasil e no exterior, atuou no Museu Nacional e como professor. Suas ideias sobre proteção à natureza ligavam-se a uma corrente de pensamento nacionalista e desenvolvimentista (Franco, Drummond, 2009, p.65 e s.).

11 Muitos deles expressavam legados sobre proteção da natureza de personalidades representativas, como Joaquim Nabuco, André Rebouças, Euclides da Cunha, Alberto Loefgren, Alberto Torres (Franco, 2002, p.78 e s.; Franco, Drummond, 2009, p.37, 65 e s.; 2005, p.132 e s.; Franco, Schittini, Braz, 2015, p.238 e s.).

12 Sobre a Conferência, que não será aprofundada aqui, ver ainda Dean (1996); Franco (2002); Franco, Drummond (2009); Franco, Schittini, Braz (2015).

13 Mesmo reconhecendo o Estado como agente condutor, Sampaio relativizava o controle e a centralização. Para ele, a responsabilidade de proteger a natureza era “transversal”, isto é, cabia a toda a sociedade e, claro, à iniciativa privada (Franco, Drummond, 2005, p.158).

14 Historicamente, a preocupação com os portos se explica pela circulação de mercadorias e pelas pessoas que, se infectadas, poderiam impactar economicamente as trocas internacionais. Sobre a questão ver Cueto (2015).

15 Sobre a questão da eugenia ver Stepan (2004) e Carvalho (2022).

16 De uso comum ou dominicais: os mares territoriais, nos mesmos incluídos os golfos, baías, enseadas e portos; as correntes, canais, lagos e lagoas navegáveis ou flutuáveis; as correntes de que se façam estas águas; as fontes e reservatórios públicos; as nascentes; os braços de quaisquer correntes públicas, desde que influam na navegabilidade ou flutuabilidade (Brasil, 1934a, artigos 1º e 2º).

17 O Serviço Florestal Federal, instituído em 1921 e regulamentado pelo decreto n.17.042, de 1925, ao qual remonta o Código Florestal, entre suas deficiências e limitações, visava proteger mananciais em áreas florestais, os quais abasteciam a cidade do Rio de Janeiro ao final dos anos 1920 (Dean, 1996, p.272).
